# Effect of apigetrin in pseudo-SARS-CoV-2-induced inflammatory and pulmonary fibrosis in vitro model

**DOI:** 10.1038/s41598-024-65447-w

**Published:** 2024-06-24

**Authors:** Hengmin Han, Jung-Eun Kim, Hyo-Jeong Lee

**Affiliations:** 1 Department of Cancer Preventive Material Development, Graduate School, College of Korean Medicine, Kyung Hee University, 26, Kyungheedae-ro, Dongdaemun-gu, Seoul, 02447 Korea; 2 Department of Science in Korean Medicine, Graduate School, College of Korean Medicine, Kyung Hee University, 26, Kyungheedae-ro, Dongdaemun-gu, Seoul, 02447 Korea

**Keywords:** SARS-CoV-2, Acute respiratory distress syndrome (ARDS), Pulmonary fibrosis (PF), Hypoxia-inducible factor-1α (HIF-1α), Apigetrin, Cell biology, Diseases

## Abstract

SARS-CoV-2 has become a global public health problem. Acute respiratory distress syndrome (ARDS) is the leading cause of death due to the SARS-CoV-2 infection. Pulmonary fibrosis (PF) is a severe and frequently reported COVID-19 sequela. In this study, an in vitro model of ARDS and PF caused by SARS-CoV-2 was established in MH-S, THP-1, and MRC-5 cells using pseudo-SARS-CoV-2 (PSCV). Expression of proinflammatory cytokines (IL-6, IL-1β, and TNF-α) and HIF-1α was increased in PSCV-infected MH-S and THP-1 cells, ARDS model, consistent with other profiling data in SARS-CoV-2-infected patients have been reported. Hypoxia-inducible factor-1 alpha (HIF-1α) siRNA and cobalt chloride were tested using this in vitro model. HIF-1α knockdown reduces inflammation caused by PSCV infection in MH-S and THP-1 cells and lowers elevated levels of CTGF, COLA1, and α-SMA in MRC-5 cells exposed to CPMSCV. Furthermore, apigetrin, a glycoside bioactive dietary flavonoid derived from several plants, including *Crataegus pinnatifida*, which is reported to be a HIF-1α inhibitor, was tested in this in vitro model. Apigetrin significantly reduced the increased inflammatory cytokine (IL-6, IL-1β, and TNF-α) expression and secretion by PSCV in MH-S and THP-1 cells. Apigetrin inhibited the binding of the SARS-CoV-2 spike protein RBD to the ACE2 protein. An in vitro model of PF induced by SARS-CoV-2 was produced using a conditioned medium of THP-1 and MH-S cells that were PSCV-infected (CMPSCV) into MRC-5 cells. In a PF model, CMPSCV treatment of THP-1 and MH-S cells increased cell growth, migration, and collagen synthesis in MRC-5 cells. In contrast, apigetrin suppressed the increase in cell growth, migration, and collagen synthesis induced by CMPSCV in THP-1 and MH-S MRC-5 cells. Also, compared to control, fibrosis-related proteins (CTGF, COLA1, α-SMA, and HIF-1α) levels were over two-fold higher in CMPSV-treated MRC-5 cells. Apigetrin decreased protein levels in CMPSCV-treated MRC-5 cells. Thus, our data suggest that hypoxia-inducible factor-1 alpha (HIF-1α) might be a novel target for SARS-CoV-2 sequela therapies and apigetrin, representative of HIF-1alpha inhibitor, exerts anti-inflammatory and PF effects in PSCV-treated MH-S, THP-1, and CMPVSC-treated MRC-5 cells. These findings indicate that HIF-1α inhibition and apigetrin would have a potential value in controlling SARS-CoV-2-related diseases.

## Introduction

Covid-19 is an infectious coronavirus disease caused by SARS-CoV-2 virus. Most SARS-CoV-2-infected individuals experience mild-to-moderate illness and recover without special treatment^1^. However, patients with advanced age, cardiovascular disease, diabetes, obesity, chronic respiratory disease, and cancer are more likely to develop severe illnesses and require medical attention. Recently, a variant mutation type of SARS-CoV-2 has emerged. SARS-CoV-2 and its variants cause short- or long-term lung complications such as pneumonia, acute respiratory distress syndrome (ARDS), sepsis, and bronchitis. Furthermore, SARS-CoV-2 leaves sequelae. The post-acute sequelae of COVID-19 (PASC) include interstitial lung disease (ILD) and pulmonary fibrosis (PF). Post-COVID-19 ILD and PF respiratory symptoms include a chronic dry cough, fatigue, and dyspnea. Ap-proximately 44.9% of COVID-19 survivors appeared to have developed PF. Factors related to COVID-19 severity were significantly associated with post-COVID-19 PF (PCPF) development.

COVID-19 has the potential to elicit a cytokine storm in pulmonary tissues, marked by immune system overactivation and unregulated cytokine release^2^ Cytokine storms can lead to a severe clinical complication called ARDS. ARDS is triggered by an exaggerated immune response rather than viral load^3^. Studies have found that extraordinarily high proinflammatory cytokine levels in SARS cytokine release syndrome, such as TNF-α, IL-6, IL-8, IL-1β, IL-12, IL-17, IFN-γ, IP10, and MCP1, are the probable causes and clinical outcomes of this phenomenon in severe COVID-19 cases and contribute to COVID-19 pathogenesis and related complications, including pulmonary inflammation and extensive lung damage^4,5^. A critical characteristic of severe COVID-19 pneumonitis is systemic low oxygen levels (hypoxaemia), which can result in organ failure and death due to acute respiratory distress syndrome (ARDS)^6,7^. Several studies showed that the up-regulation and participation of hypoxia-inducible factor-1α (HIF-1α) relate to inflammation, immunometabolism, and TLR in bronchoalveolar cells of critically severe COVID-19 patients^8^. Also, high HIF-1α levels were detected in peripheral blood monocytes of COVID-19 patients and infected cells^9^. Hypoxia induces HIF-1alpha, a transcription factor that regulates various genes involved in physiological functions such as, inflammation, cancer metabolism, cell proliferation, and angiogenesis^10–14^. When oxygen is plentiful, newly produced HIFα subunits are hydroxylated by HIF prolyl-hydroxylase domain (PHD) enzymes, leading to their breakdown by the proteasome. In hypoxic conditions, PHD enzymes become inactive, allowing stabilized HIF-1α to dimerize with HIF-1β. This complex moves to the nucleus and activates the transcription of genes related to erythropoiesis, glycolysis, pulmonary vasomotor control, and immune regulation^12,15,16^. HIF-1α is also an important activator in glycolysis and inflammatory response, which implies the effects of HIF-1α on the pathogenesis of COVID-19^17,18^. Therefore, it is essential to comprehend the pathogenesis of SARS-CoV-2 and explore potential antiviral treatments.

Recently, many studies have reported the development of SARS-CoV-2 vaccines and drugs. However, most targeted therapeutics against SARS-CoV-2 focus on vaccines or blockers of entry into cells to prevent SARS-CoV-2 infection. Supportive therapeutic interventions and antiviral agents with limited efficacy are required to reduce the COVID-19 outbreak and mitigate its symptoms^19,20^. Although much research has focused on developing vaccines and antiviral therapies to prevent or treat SARS-CoV-2 infection, studying their long-term effects and post-infection complications is crucial. Few studies have targeted inflammation (immunomodulatory properties). Few studies have investigated targeted therapeutic agents for post-SARS-CoV-2-related lung diseases. Furthermore, most SARS-CoV-2 experiments are difficult to perform in a laboratory without biosafety level 3 (BSL-3) permission. Therefore, a reliable, rapid, and convenient in vitro model of SARS-CoV-2-related lung disease that can be handled in a BSL-2 laboratory is essential for screening and evaluating therapeutic agents against SARS-CoV-2 infection. Therefore, based on SARS-CoV-2 clinical data reported, we established in vitro models of ARDS and PF using pseudotyped SARS-CoV-2 virus, and inflammation and PF-related markers were assessed in this study.

Natural products have received considerable attention as potential therapeutic options. Flavonoids, naturally occurring polyphenolic compounds in fruits and vegetables, have received significant attention owing to their anticancer, anti-inflammatory, antioxidant, antibacterial, fungal, and viral properties. Therefore, flavonoid-rich flavonoids and plants are considered applicable agents for treating COVID-19, and their potentially beneficial role is currently a widely discussed topic. Apigetrin, apigenin 7-O-glucoside, and cosmosin are flavonoid glycosides commonly found in natural sources. Apigenin has various beneficial properties including antioxidant, anti-obesity, anticancer, and anti-inflammatory effects^21–25^. We have determined that apigetrin has an anti-cancer effect by inhibiting HIF-1α^26^. Therefore, based on the reported data on apigetrin, we evaluated the effect of apigetrin on SARS-CoV-2 complications mimicking in vitro models.

## Materials and methods

### Test chemical

Apigetrin (M.W = 432.38 g/mol, purity ≥ 97% as determined through HPLC) was purchased from Sigma-Aldrich (Cat: 44692, St. Louis, MO, USA). HIF-1α siRNA was purchased from Santa Cruz (Cat: sc-35561, Dallas, TX, USA), and CoCl_2_ was purchased from Sigma-Aldrich (Cat: C8661, St. Louis, MO, USA).

### Cell culture

MH-S (Cat: CRL-2019, American Type Culture Collection (ATCC), Manassas, VA, USA) and THP-1 cells (Cat: TIB-202, ATCC, Manassas, VA, USA) were cultured in RPMI 1640 (Cat: LM 011-01, Welgene, Daegu, Korea) medium supplemented with 10% fetal bovine serum (Cat: S101-07, Welgene, Daegu, Korea) and 1% antibiotics (Cat: LS203-01, Welgene, Daegu, Korea). Lenti-X 293T (Cat: 632180, Takara Bio Inc., Shiga, Japan) and MRC-5 (Cat: 10171, KCLB, Seoul, Korea) cells were cultured in DMEM (Cat: LM-001-05, Welgene, Daegu, Korea) medium supplemented with 10% FBS and 1% antibiotics. All the cells were cultured in a humidified atmosphere at 37 °C containing 5% CO_2_ to maintain the required physiological conditions.

### SARS-CoV-2 pseudo-typed lentivirus

Lenti-X 293T cells (5 × 10^6^) were seeded/10 cm plate in complete medium for 24 h. The Lenti-X SARS-CoV-2 Packaging single shots-Wuhan-Hu-1 (Cat: 632668, Takara, Vancouver, Canada) was used to cultivate the SARS-CoV-2 pseudo typed lentivirus. After 4 h treating Lenti-X SARS-CoV-2 Packaging Single Shots, 6 mL of fresh complete growth medium was added and incubated at 37 °C, 5% CO_2_ for an additional 48 h. 5 × 10^7^ pseudo-SARS-CoV-2 were used to infect MH-S and THP-1 cells.

### Immunofluorescence

MH-S and THP-1 cells (1 × 10^4^ cells) were seeded in confocal dishes (Cat: 210350; SPL, Pocheon, Korea). After, the cells were incubated for 24 h at 37 °C containing 5% CO_2_, treated with PSCV and apigetrin (10 μM) for 24 h, then were fixed in 100% methanol (chilled at − 20 °C) at room temperature for 5 min. The fixed cells were exposed to the diluted Spike 1 (Cat: 99423, Cell Signaling, Danvers, MA, USA) antibody in 1% BSA in PBST in a humidified chamber overnight at 4 °C. Then, cells were incubated with the goat anti-rabbit IgG (Alexa Fluor 488) (Cat: ab150077, Abcam, Cambridge, UK) in 1% BSA for 1 h at room temperature in the dark. The reacted cells were incubated with VECTASHIELD antifade mounting medium with DAPI (Cat: H-1200-10, Vector Laboratories, Burlingame, CA, USA) for 3 min. Images were captured randomly and each slide was imaged using a fluorescence microscope (Nikon, Tokyo, Japan).

### SARS-CoV-2 binding assay

A SARS-CoV-2 inhibitor screening kit (Cat: EP-105, Acrobiosystems, Newark, DE 19711, USA) was used to test the ability of apigetrin to bind to the SARS-CoV-2 S protein RBD. SARS-CoV-2 S protein RBD stock solution (100 μg/mL) was diluted to 0.5 μg/mL with a coating buffer to make the SARS-CoV-2 S protein RBD working solution. Biotinylated human ACE2 stock solution (100 μg/mL) was diluted to 0.12 μg/mL with a dilution buffer to make a biotinylated human ACE2 working solution. A total of 50 μL of apigetrin (10 μM) was added, and the plate was sealed with a microplate sealing film and incubated for 1 h. Streptavidin-HRP stock solution (50 μg/mL) was diluted to 0.1 μg/mL with a dilution buffer and incubated for 1 h. Then, 100 μL of Streptavidin-HRP working solution was added and incubated for 1 h. TMB Substrate Working Solution (200 μL) was added to each well and incubated for 20 min. Then, 50 μL of stop solution was added and tapped gently for 3 min. The absorbance was measured at 450 nm using a UV microplate spectrophotometer.

### Western blot analysis

Protein samples were obtained by lysing cells in radioimmunoprecipitation assay (RIPA) buffer, which comprised 50 mM Tris–HCl at pH 7.4, 150 mM NaCl, 1% NP-40, 0.25% sodium deoxycholate, 1 M EDTA, 1 mM Na_3_VO_4_, 1 mM NaF, and protease inhibitor cocktail (Cat: 89900, 87786, 78420, Thermo, Waltham, MA, USA). Protein concentration was determined using the Bio-Rad DC Protein Assay Kit II (Cat: 500-0113, 500-0114, 500-0115, Bio-Rad, Hercules, CA, USA). The quantified protein samples were loaded onto 8–15% sodium dodecyl sulfate-polyacrylamide gel electrophoresis (SDS-PAGE) gels and transferred onto a Hybond transfer membrane (Cat: 10600002, 10600001, Amersham Pharmacia, Piscataway, NJ, USA) using a transfer system at 250 mA for 2 h. Non-specific binding on membranes was blocked in fresh 5% non-fat dry milk (Cat: DI-232100, BD, Franklin Lakes, NJ, USA), after which the membranes were incubated with primary antibodies against IL-6 (ratio 1:2000, Cat: SC-28343, Santa Cruz, Dallas, TX, USA), Spike1 (ratio 1:1000, Cat: 99423, Cell Signaling, Danvers, MA, USA), IL-1β (ratio 1:1000, Cat: SC-12742, Santa Cruz, Dallas, TX, USA), TNF-α (ratio 1:1000, Cat: SC-52746, Santa Cruz, Dallas, TX, USA), CTGF (ratio 1:1000, Cat: SC-101586, Santa Cruz, Dallas, TX, USA), COLA1 (ratio 1:1000, Cat: ab6308, Abcam, Cambridge, UK), α-SMA (ratio 1:1000, Cat: SC-53015, Santa Cruz, Dallas, TX, USA), HIF-1α (ratio 1:500, Cat: NB100-105, NOVUS, Littleton, CO, USA), and β-actin (ratio 1:10,000, Cat: A5316, Sigma-Aldrich, St. Louis, MO, USA) overnight at 4 °C. Membranes were incubated with horseradish peroxidase (HRP)-conjugated anti-mouse (ratio 1:10,000, Cat: 115-035-003; Jackson, West Grove, PA, USA) or anti-rabbit secondary anti-bodies (ratio 1:10,000, Cat: 111-035-003, Jackson, West Grove, PA, USA). An enhanced chemiluminescence (ECL) system (Cat: RPN2209; Amersham Pharmacia, Piscataway, NJ, USA) was used for the protein expression analysis. Densitometric data were obtained by quantifying each protein band using ImageJ 1.53k (National Institutes of Health, Bethesda, MD, USA). Protein levels were quantified from the duplicate analyses of each sample. Protein levels of interest were normalized to β-actin.

### SARS-CoV-2 cytokines secretion quantification

MH-S and THP-1 cells (1 × 10^6^) were seeded in a 60 mm cell culture dish and incubated for 24 h. The cells were treated with or without PSCV and apigetrin (10 μM) for 24 h, then the supernatant was harvested. The secretion of TNF-α, IL-6, and IL-1β by MH-S or THP-1 cells was measured using ELISA with a paired set of antibodies and HRP-labeled streptavidin (Cloud-Clone, Cat: SEA079Hu, SEA133Hu, SEA563Hu, SEA563Mu, SEA133Mu, SEA079Mu, Katy, TX, USA). The optical density was measured at 450 nm using a microplate reader (Sunrise RC, Tecan, Mannedorf, Switzerland).

### Cell proliferation assay

MRC-5 cells (1 × 10^4^) were seeded in 96-well plates (Cat: 30096; SPL, Pocheon, Korea) and incubated for 24 h. The cells were treated with or without apigetrin (10 μM) in CMPSCV for 24 h. Cell viability was evaluated using a CELLOMAX viability kit (Cat: CM-VA2500, Precaregene, Hanam, Korea). CELLOMAX reagent (10 μL) was added to each well and was incubated in the dark. The optical density was measured at 450 nm using a microplate reader (Sunrise RC, Tecan, Mannedorf, Switzerland).

### Migration assay

MRC-5 cells (5 × 10^4^) were seeded into a Cell Culture Insert (Cat: 36224, SPL, Pocheon, Korea) at a density of 2.5 × 10^5^/mL in a serum-free medium. 700 μL of CMPSCV or CTPSCV medium (with serum) treated with or without apigetrin in the lower chamber. Transfer the insert into the lower chamber. The plates were incubated for 24 h at 37 °C containing 5% CO_2_. The medium was removed, and the cells were washed with PBS. Cells were fixed with 3.5% formaldehyde (in PBS) at room temperature for 2 min and permeabilized with methanol at room temperature for 20 min. The cells were then stained with crystal violet at room temperature for 15 min. Nonmigratory cells were scraped off using cotton swabs. Images were captured under a light microscope (Nikon, Tokyo, Japan).

### Statistical analysis

The data presented in this study are expressed as mean ± standard deviation (S.D.) and were obtained from three replicates for each experiment. Analysis of variance was used to assess the significance of differences between groups. A p-value less than 0.05 was considered statistically p < 0.05. Significance was evaluated using the Sigma Plot software (*p < 0.05, **p < 0.01, ***p < 0.001). Data were analyzed by one-way ANOVA, followed by Tukey’s studentized range test using the GraphPad Prism 8.0 software (GraphPad Software Inc., San Diego, CA, USA). Means with different letters are significantly different between the groups.

## Results

### Mimicry ARDS in vitro model using PSCV in MH-S and THP-1 cells shows increased proinflammatory cytokines and HIF-1α

**Figure 1:**
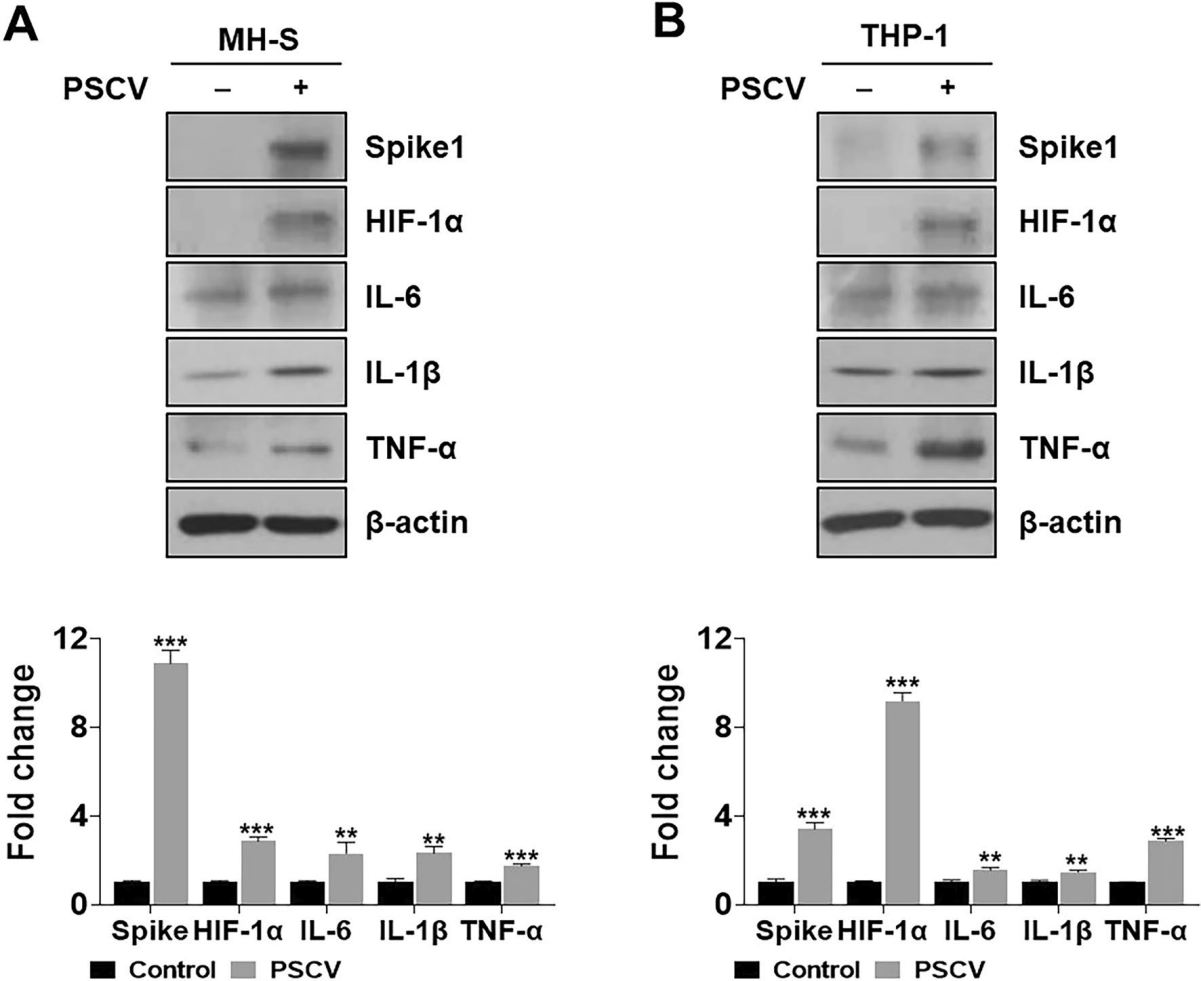
Evaluation of in vitro ARDS model. MH-S (**A**) and THP-1 cells (**B**) were infected with PSCV for 24 h. Cell lysates were prepared and subjected to western blotting to analyze the expression of Spike1, HIF-1α, IL-6, IL-1β, TNF-α, and β-actin. The results are presented as the mean ± SD of three independent experiments. **p < 0.01, and ***p < 0.001 (compared to normal control).

Lipopolysaccharide-treated MH-S cells were used as an in vitro model of ARDS^27–34^. We modified this model to establish an in vitro model that mimics ARDS caused by SARS-CoV-2. An in vitro model, a SARS-CoV-2 ARDS mimic, was produced by treating THP-1 and MH-S cells with PSCV instead of LPS. We checked the proinflammatory cytokines and HIF-1α levels that have been reported as highly expressed in SARS-CoV-2 patients. As shown in Fig. 1A,B, IL-6, IL-1β, TNF-α, and HIF-1α showed over 1.5-fold higher expression than PSCV-non-treated cells in MH-S and THP-1 cells. Especially, HIF-1α expression indicated 2.8- and 9.2-fold higher expression than PSCV-non-treated cells in, respectively, MH-S and THP-1 cells.

### Mimicry PF in vitro model using a conditioned medium of PSCV-treated MH-S or THP-1 cells (CMPSV) in MRC-5 cells shows increased expression of CTGF, COLA1, α-SMA, and HIF-1α

**Figure 2:**
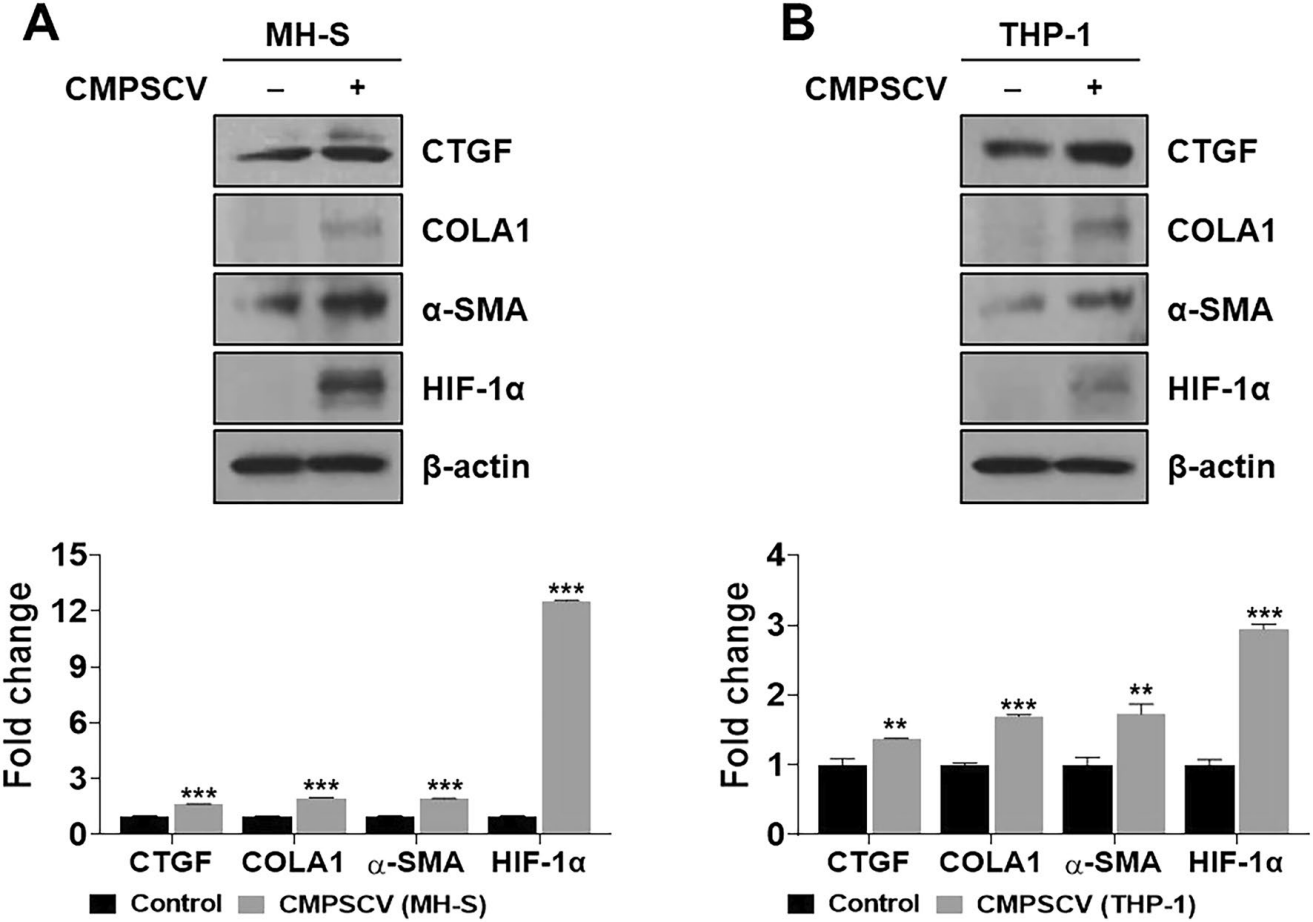
Evaluation of in vitro PF model. MRC-5 cells were treated with CMPSCV from MH-S (**A**) or THP-1 (**B**) cells for 24 h. Cell lysates were prepared and subjected to western blotting to analyze the CTGF, COLA1, α-SMA, HIF-1α, and β-actin expressions. The results are presented as the mean ± SD of three independent experiments. **p < 0.01, and ***p < 0.001 (compared to normal control).

PF is frequently reported as a COVID-19 sequela of^35–38^. As elevated cytokine levels regulate the initiation and progression of fibrosis and remodeling, an in vitro model mimicking SARS-CoV-2 PF was produced by treating MRC-5 cells with CMPSCV. CMPSCV is a culture medium obtained after a 48-h culture of culturing PSCV-infected MH-S or THP-1 cells. The in vitro PF model was established by culturing MRC-5 cells in the two types of CMPSCV for 24 h. If CMPSCV is involved in MRC-5 differentiation to change fibroblast to myofibroblast, we expected MRC-5 cells to increase CTGF, COLA-1, and α-SMA. As shown in Fig. 2A,B, CTGF, COLA1, α-SMA, and HIF-1α expression significantly increased in CMPSCV of MH-S or THP-1 cells compared to the control group, without CMPSCV (Supplementary Figures).

### HIF-1α knockdown ameliorates inflammation induced by PSCV infection in MH-S and THP-1 cells and decreases increased CTGF, COLA1, and α-SMA in CPMSCV-exposure MRC-5 cells

**Figure 3:**
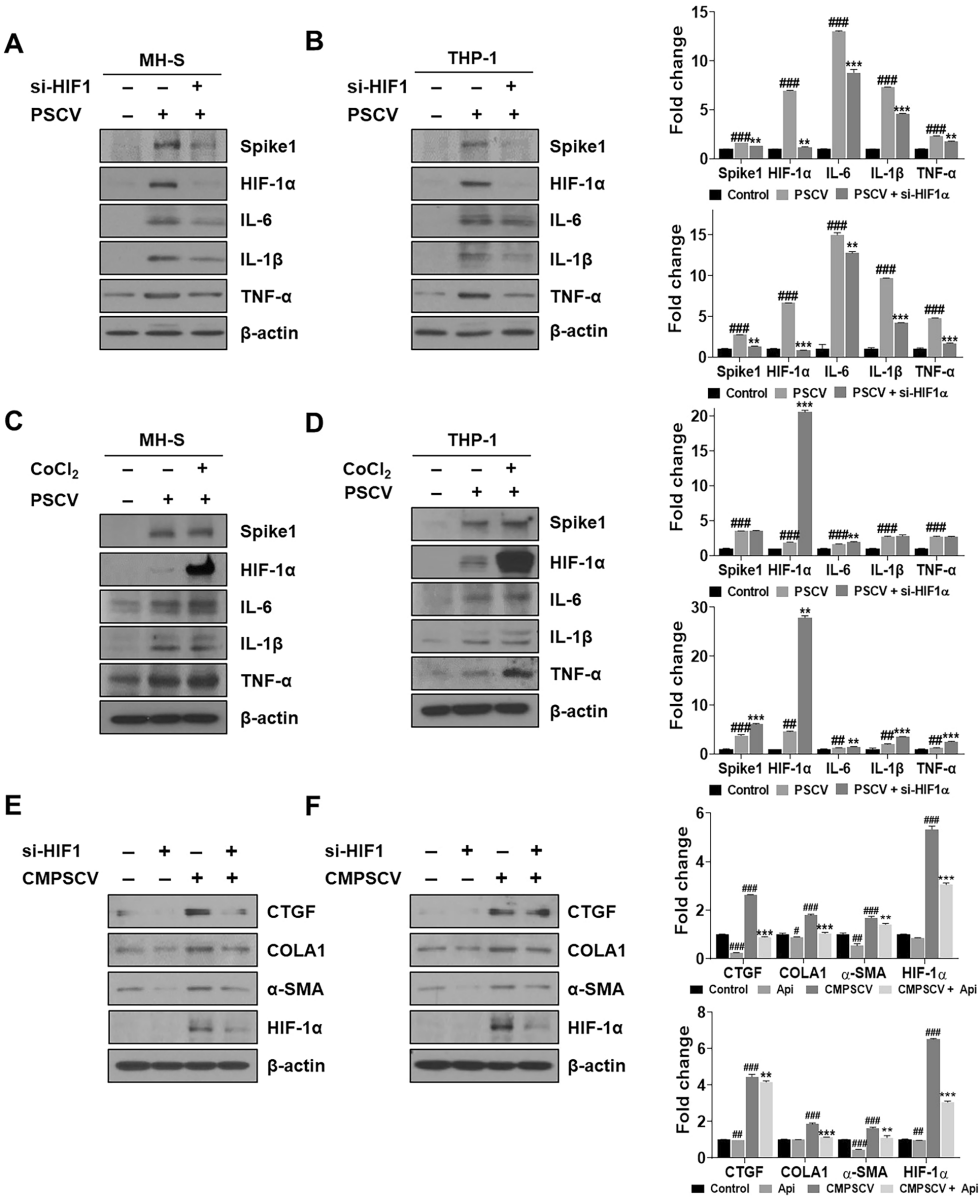
Effect of HIF-1α siRNA and CoCl_2_ in PSCV-infected MH-S or THP-1 cells and CPMSCV-exposure MRC-5 cells. MH-S (**A**) and THP-1 cells (**B**) were transfected with HIF-1α siRNA for 48 h and were incubated in the presence or absence of PSCV for 24 h. PSCV-infected MH-S (**C**) and THP-1 cells (**D**) were treated with CoCl_2_ for 24 h. Cell lysates were prepared and subjected to western blotting to analyze the Spike1, HIF-1α, IL-6, IL-1β, TNF-α, and β-actin expressions. The results are presented as the mean ± SD of three independent experiments. ^*##*^*p* < 0.01, ^###^*p* < 0.001 (compared to normal control), **p < 0.01, and ***p < 0.001 (compared to PSCV-infected control). MRC-5 cells were transfected with HIF-1α siRNA for 48 h and were incubated in the presence or absence of PSCV-treated MH-S (**E**) or THP-1 cells (**F**) CMPSCV for 24 h. Cell lysates were prepared and subjected to western blotting to analyze the CTGF, COLA1, α-SMA, HIF-1α, and β-actin expressions. The results are presented as the mean ± SD of three independent experiments. ^##^*p* < 0.01, ^###^*p* < 0.001 (compared to normal control), **p < 0.01, and ***p < 0.001 (compared to CMPSCV-infected control).

To confirm the role of HIF-1α in the PSCV-induced inflammation in MH-S and THP-1 cells, HIF-1α knockdown experiments were performed. HIF-1α protein 6.9 and 6.64 folds higher expressed in PSCV-infected MH-S and THP-1 cells compared with non-infected MH-S and THP-1 cells, while HIF-1α siRNA blocked the PSCV-induced HIF-1α protein expression in MH-S and THP-1 cells. Also, HIF-1α siRNA suppressed inflammatory proteins IL-6, IL-1β, and TNF-α levels increased in PSCV-infected MH-S and THP-1 cells (Fig. 3A,B). Spike protein showed strong expression in PSCV-infected MH-S cells and THP-1 cells, while the spike protein was decreased by treating HIF-1α siRNA to PSCV-infected MH-S and THP-1 cells (Fig. 3A,B). In contrast, cobalt chloride (CoCl_2_), a hypoxia mimic and inhibitor of HIF-1α degradation, induced inflammatory cytokines in the PSCV-exposure THP-1 cells. In PSCV-treated MH-S cells, CoCl_2_ maintained the levels of cytokines that were increased by PSCV (Fig. 3C,D). HIF-1α siRNA decreased increased CTGF, COLA1, α-SMA, and HIF-1α in CMPSCV-cultured MRC-5 cells (Fig. 3E,F).

### Apigetrin obstructs entering SARS-CoV-2 into the cells

**Figure 4:**
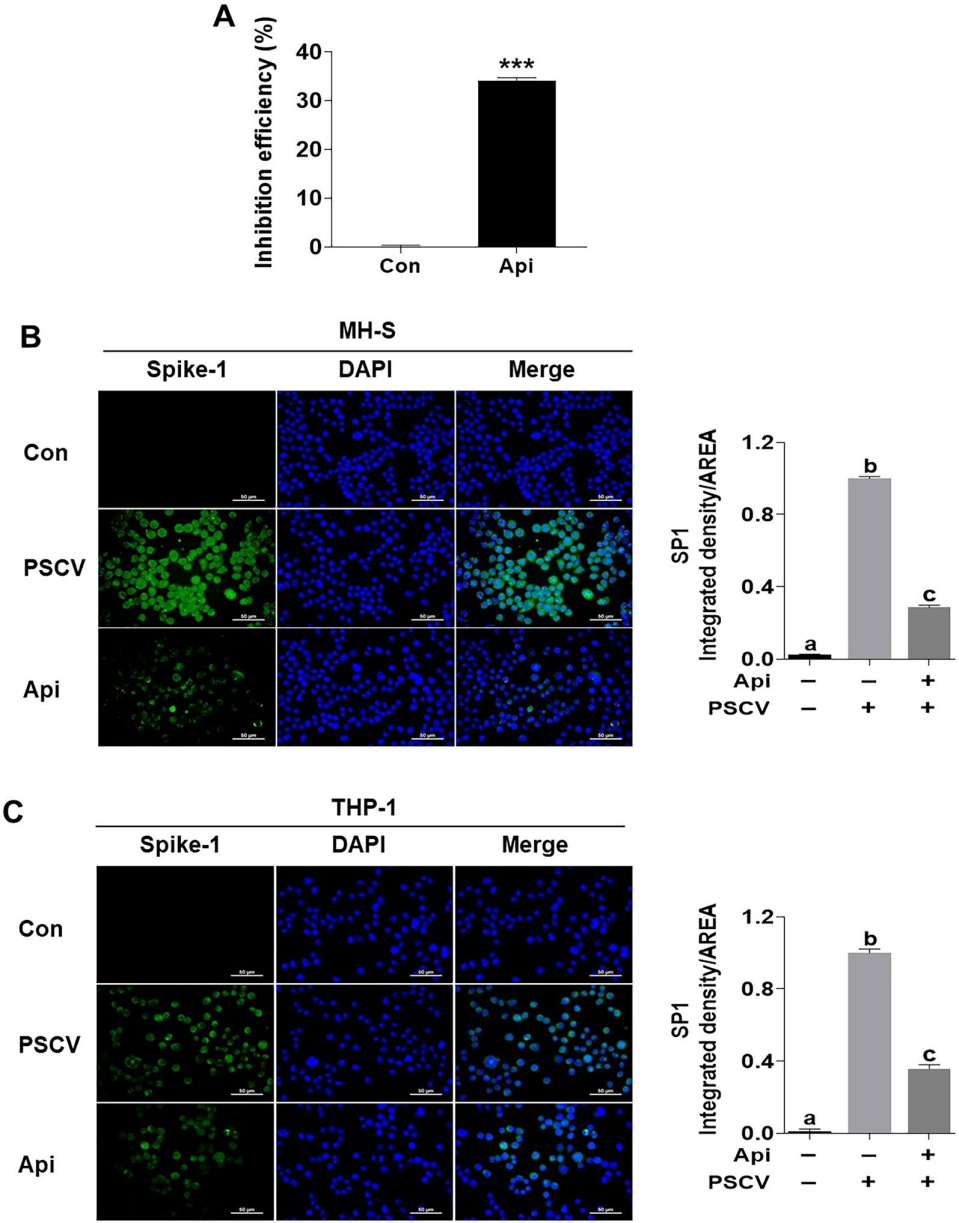
Evaluation of apigetrin on spike protein expression and binding to S protein RBD in PSCV-infected MH-S and THP-1 cells. (**A**) The SARS-CoV-2 inhibitor screening kit was used to test the ability of apigetrin to bind to SARS-CoV-2 S protein RBD. The results are presented as the mean ± SD of three independent experiments. ***p < 0.001 (compared to normal control). MH-S (**B**) and THP-1 cells (**C**) were treated with PSCV and apigetrin for 24 h. The cells were fixed in 100% methanol, exposed to the diluted Spike 1 antibody, and incubated with the goat anti-rabbit IgG. The reacted cells were incubated with VECTASHIELD antifade mounting medium with DAPI, and the image was captured using a fluorescence microscope. a–c means in a row by different superscripts are significantly different by LSD (least significant difference).

The initial attachment of SARS-CoV-2 to cells involves specific binding between the viral spike (S) glycoprotein and the cellular receptor ACE2. To evaluate whether apigetrin interferes with the formation of the S-ACE2 complex, a SARS-CoV-2 S-ACE2 binding ELISA kit was used. Apigetrin displayed 34% blocking activity between the binding spike S RBD: ACE2 receptor (Fig. 4A). Additionally, we checked the spike protein in the cells after infection with PSCV to MH-S and THP-1 cells. As shown in Fig. 4B,C, the spike protein was highly expressed in PSCV-infected MH-S and THP-1 cells. Spike protein expression decreased in PSCV-infected MH-S and THP-1 cells treated with apigetrin. The spike protein expression of apigetrin was reduced by 3.3 and 2.8 folds, respectively, compared with the PSCV-treated MH-S and THP-1 cells (Fig. 4A).

### Apigetrin inhibited the inflammatory effect induced by PSCV infection in MH-S and THP-1 cells

**Figure 5:**
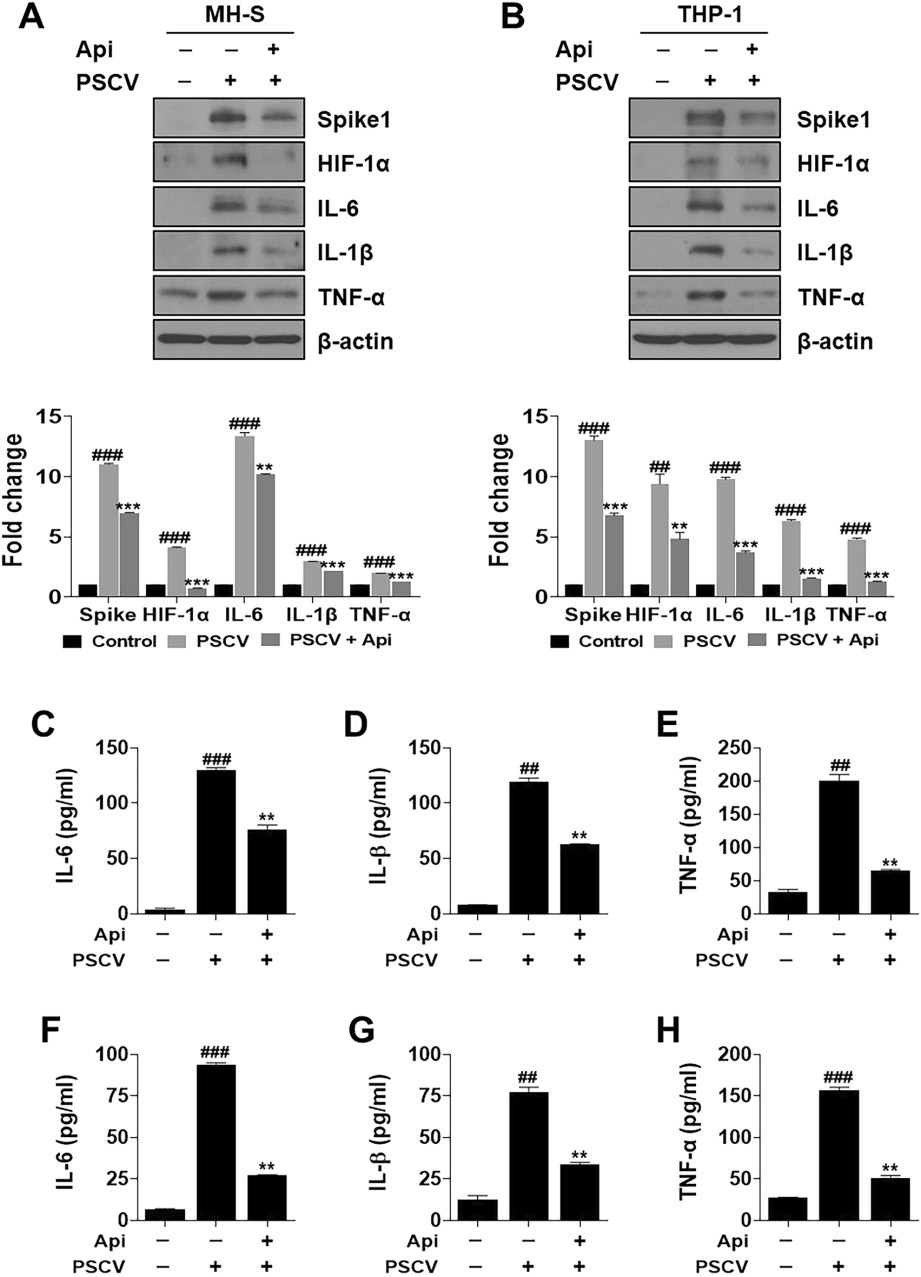
Anti-inflammatory effect of apigetrin in PSCV-infected MH-S and THP-1 cells. MH-S (**A**) and THP-1 cells (**B**) were infected with PSCV and apigetrin for 24 h. Cell lysates were prepared and subjected to western blotting to analyze the Spike1, HIF-1α, IL-6, IL-1β, TNF-α, and β-actin expressions. IL-6, IL-1β, and TNF-α concentrations in the serum samples of MH-S (**C**–**E**), and THP-1 (**F**–**H**) cells were determined by ELISA. The results are presented as the mean ± SD of three independent experiments. ^##^*p* < 0.01, ^###^*p* < 0.001 (compared to normal control), **p < 0.01, and ***p < 0.001 (compared to PSCV-infected control).

PSCV-infected MH-S or THP-1 cells showed an increase over two-fold in inflammatory relative cytokines (IL-6, IL-1β, and TNF-α) and spike protein levels compared with non-infected cells (Fig. 5A,B). In contrast, apigetrin attenuated the PSCV-induced increase in inflammatory cytokines and spike protein levels (Fig. 5A,B). IL-6, IL-1β, and TNF-α secretion levels increased by PSCV in MH-S and THP-1 cells (Fig. 5C–H). The amount showed 129.5 pg/mL for IL-6, 119 pg/mL for IL-1β, and 201 pg/mL for TNF-α increasing 34.54, 14.4, and 6.19 folds respectively, compared with the control group (3.75, 8.23, and 32.3 pg/mL) in MH-S cells (Fig. 5C–E). Apigetrin reduced the increased IL-6, IL-1β, and TNF-α secretion levels up to 22.5, 47.3, and 68.2%, respectively (Fig. 5C–E). In the case of THP-1 cells, the increased amount indicated 93.9, 77.29, and 156 pg/mL for IL-6, IL-1β, and TNF-α, rising 14, 6.08, and 5.71 folds by PSCV, respectively, compared with the control group (6.7, 12.7, and 27.3 pg/mL) (Fig. 4F–H). Similar to MH-S cell data, apigetrin decreased the increased secretion levels of cytokines up to 71, 56.6, and 67.2% for IL6, IL-1β, and TNF-α, respectively (Fig. 5F–H).

### Apigetrin attenuated MRC-5 cell proliferation, migration, and expression of CTGF, COLA1, α-SMA, and HIF-1α induced by conditioned medium from PSCV-treated MH-S or THP-1 cells (CMPSCV)

**Figure 6:**
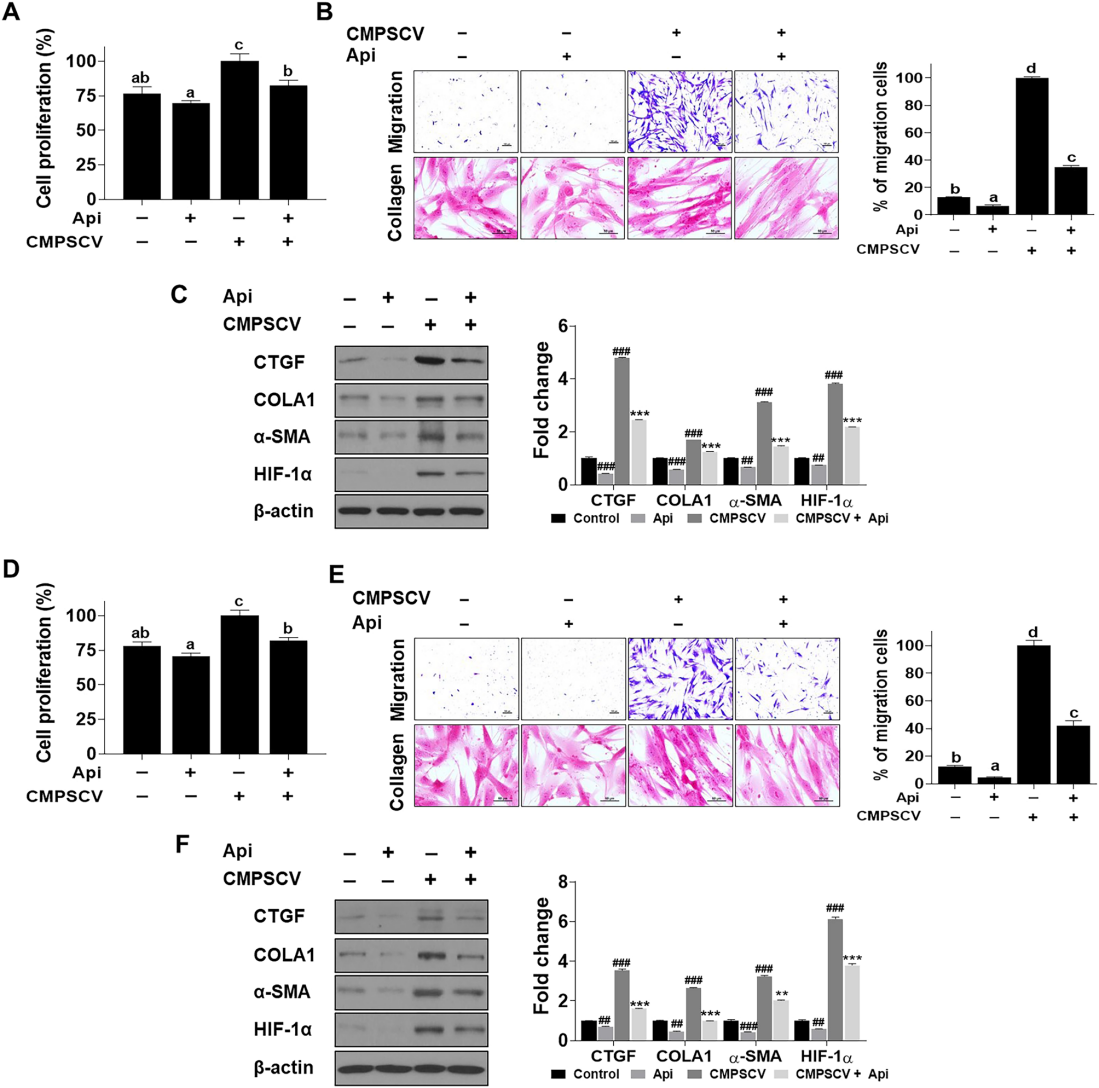
Effect of apigetrin on pulmonary fibrosis in CMPSCV-treated MRC-5 cell model. MRC-5 cells were treated with apigetrin in MH-S (**A**,**B**) or THP-1 cells (**D**,**E**) CMPSCV for 24 h. CELLOMAX kit was used to measure cell proliferation. a–c means in a row by different superscripts are significantly different by LSD (least significant difference). After treatment the cells were resolved in 3.5% formaldehyde, permeabilized by methanol, and stained by crystal violet. Picro-Sirius Red Stain Kit was used to stain collagen fibers and the image was captured using a light microscope (**B**,**E**). a–d means in a row by different superscripts are significantly different by LSD (least significant difference). MRC-5 cells were treated with apigetrin in MH-S (**C**) or THP-1 cells (**F**) CMPSCV for 24 h. Cell lysates were prepared and subjected to western blotting to analyze the CTGF, COLA1, α-SMA, HIF-1α, and β-actin expressions. The results are presented as the mean ± SD of three independent experiments at *p* < 0.05. ^##^*p* < 0.01, ^###^*p* < 0.001 (compared to normal control), **p < 0.01, and ***p < 0.001 (compared to CMPSCV-infected control).

The effect of apigetrin on PSCV-induced PF was evaluated in CMPSCV-induced MRC-5 cells. Apigetrin reduced the MRC-5 cell proliferation by 18% CMPSCV of MH-S and THP-1 cells enhanced (Fig. 6A,D). Transwells were used to assess the dynamic fibroblast migration in PF. CMPSCV of MH-S or THP-1 cells increased MRC-5 cell migration by 88 and 87%, respectively, compared to non-treated cells (Fig. 6B,E). The increase in MRC-5 cell motility by CMPSCV was suppressed by apigetrin by 65% and 58%, respectively. Apigetrin significantly decreased the increased CTGF, COLA1, α-SMA, and HIF-1α expression by CMPSCV (Fig. 6C,F). Picro Sirius red staining was used to assess collagen expression and observe the differentiated phenotype of MRC-5 cells in response to CMPSV. As shown in Fig. 6B,E, CMPSCV in MH-S or THP-1 cells induced long and stiff-type cells and darker MRC-5 cells. Apigetrin induced a shorter and lighter cell morphology than CMPSCV-induced MRC-5 cells (Fig. 6B,E).

## Discussion

In this study, we focused on SARS-CoV-2 post-infection complications and established in vitro models to mimic SARS-CoV-2 infection-related complications. Many studies have reported on the development of SARS-CoV-2 vaccines and drugs that block entry into cells to prevent SARS-CoV-2 infection. According to recently reported clinical data, ACE2 inhibitors and angiotensin receptor blockers (ARBs), the leading SARS-CoV-2 treatments, have not improved clinical outcomes in adult patients with severe COVID-19 and may even worsen them^39^. The renin-angiotensin system (RAS) relates to SARS-CoV-2 infection, ARDS, pulmonary hypertension, and PF^40,41^ RAS system comprises two pathways. One is the angiotensin II (Ang II) generation from angiotensinogen by the enzymes renin and ACE. Ang II binds to the angiotensin type 1 receptor (AT1R) to cause tissue in-jury such as pulmonary hypertension and fibrosis. Another pathway involves the degradation of Ang II to angiotensin 1–7 by the enzyme ACE2, which protects against tissue damage. Therefore, ACE2 plays a counterregulatory role in inhibiting the RAS by removing Ang II. ACE2 has been reported to prevent the development of severe acute respiratory syndrome in SARS-CoV infection^42^ and protect against severe acute lung failure^43^, it has been reported that ACE2 plays a functional receptor on cell surfaces through which SARS-CoV-2 enters host cells^44,45^. In summary, ACE2 is a double-edged sword. There-fore, these targeted inhibitors appear to be suitable for the early or preventive treatment of SARS-CoV-2. ARDS and PF are serious SARS-CoV-2 infection complications. Investigating immunomodulatory therapies is essential, as an overactive immune response (cytokine storm) has been implicated in severe COVID-19 cases. Targeting inflammation and immune responses may help manage post-SARS-CoV-2 complications. To date, the well-known drugs for SARS-CoV-2 immune hyperactivity include tyrosine kinase inhibitors of JAK-STAT signaling and glucocorticoids. Although dexamethasone and methylprednisolone are effective glucocorticoids in reducing the severity of COVID-19 and associated comorbidities such as chronic obstructive pulmonary diseases^46^, these glucocorticoid drugs represent a double-edged sword in COVID-19 therapy. The long-term use of high-dose glucocorticoids causes numerous side effects, such as fluid retention, weight gain, and hyperglycemia^47^. JAK inhibitors have been clinically used to treat several inflammatory and autoimmune diseases^48,49^. Therefore, JAK inhibitors have been proposed as pharmacological therapies for COVID-19. Tofacitinib, imatinib, and baricitinib exerted clinical benefits in hospitalized patients with COVID-19; however, further studies are required to validate these findings.

Immunological dysregulation, also known as the “cytokine storm” by SARS-CoV-2, may be a significant contributor to multiorgan dysfunction^50,51^. Many cytokines have been reported at elevated levels in COVID-19 cases, including IL1-β, IL-6, IL-7, IL-8, TGF-β and TNF-α. Elevated pro-inflammatory cytokines contribute to COVID-19-related complications such as ARDS or PF, mediating the initiation and progression of fibrosis and remodeling^52,53^. Based on these studies, we generated in vitro ARDS and PF models to mimic SARS-CoV-2 complications using pseudo-SARS-CoV-2 in MH-S, THP-1, and MRC-5 cells. Therefore, developing reliable and convenient in vitro models is a valuable approach. These models enable researchers to study disease mechanisms and test potential therapeutic agents in a controlled environment. Using pseudotyped SARS-CoV-2 can help simulate viral infections without the need for a high-containment BSL-3 laboratory, making it more accessible for research. To ensure that our in vitro model closely mimics the clinical aspects of post-SARS-CoV-2 complications, we checked the proinflammatory cytokines, PF-related markers, and HIF-1α levels that have been reported as highly expressed in SARS-CoV-2 patients in PSCV- exposure MH-S and THP-1 cells or CPMSCV-cultured MRC-5 cells. As shown in Fig. 1A,B, IL-6, IL-1β, TNF-α, and HIF-1α showed higher expression than PSCV-non-treated cells in MH-S and THP-1 cells. A conditioned medium of PSCV-treated MH-S or THP-1 cells (CMPSV) in MRC-5 cells shows increased CTGF, COLA1, α-SMA, and HIF-1α expressions in Fig. 2A,B.

HIF-1α is recognized as a significant activator in glycolysis and is also implicated in inflammatory responses. This suggests that HIF-1α may play a role in the pathogenesis of COVID-19^17,18^. A recent paper has reported that the HIF-1α mRNA level is much higher in PBMCs of COVID-19 inflammatory patients related to healthy individuals ^9^. Also, the contribution of hypoxia and HIF-1α to the development of interstitial PF as well as PF caused by SARS-CoV-2 is already known^54–57^. Consistent with other reported COVID-19 clinical data, HIF-1α levels increased in PSCV-infected MH-S and THP-1 cells (Fig. 1), as well as in CMPSV-stimulated MRC-5 cells (Fig. 2). In contrast, HIF-1α knockdown was shown to ameliorate inflammation induced by PSCV infection in MH-S and THP-1 cells and decrease increased CTGF, COLA1, and α-SMA in CPMSCV-exposure MRC-5 cells (Fig. 3A,B,E,F). Cobalt chloride (CoCl_2_), a hypoxia mimic, increased or maintained the cytokine levels induced by PSCV in MH-S and THP-1 cells (Fig. 3C,D).

On the contrary, recently, starting with epidemiological reports for lower COVID-19 incidence in populations living at high altitudes, a few studies supporting the epidemiological reports interestingly showed that an association of the rs11549465 variant of HIF1-α with COVID-19 susceptibility was recently discovered^58^ and hypoxic and pharmaco-logical drug of HIF inhibit the infection of lung cells by SARS-CoV-2 by reducing ACE2 viral receptor expression^59^. However, while these studies are sufficient to help us understand the molecular mechanisms behind the lower incidence of COVID-19 in high-altitude populations, they do not help explain “happy hypoxia,” a pathophysiological phenomenon in which hypoxia increases a patient’s risk of developing a severe form of COVID-19^60^. Methods for targeting ACE2 can induce the possible dysfunction of signals when ACE2 is reduced, makes Ang(1–7) no longer synthesized, and AngII accumulates and binds AT1R, leading to HIF-1α causing tissue injury^61,62^. This is also why SARS-CoV-2 binds to ACE2, allowing it to enter cells and cause more severe disease. Furthermore, in the recently reported study by Peter A.C. Wing et al., hypoxia and FG-4592 increased ACE, but the expression of mRNA for HIF-1alpha, a known hypoxia marker, was reduced in these treated cells than in normoxia, 18% O_2_ levels^59^. Unlike the recently reported study by Peter A.C. Wing et al., it has been reported that hypoxia increases ACE, ACE2, and HIF-1α expression in pulmonary artery smooth muscle cells (PASMCs)^40^ and pulmonary artery adventitial fibroblasts (FBPA)^54^, and HIF-1siRNA decreases ACE expression and keeps ACE2 expression in normoxic-PASMCs^31^. CoCl_2_, a hypoxia mimic, maintained or induced spike protein expression in PSCV-stimulated MH-S and THP-1 cells (Fig. 3C,D). However, HIF-1α siRNA reduces spike protein expression in PSCV-stimulated MH-S and THP-1 cells (Fig. 3A,B). As shown in Fig. 4C, we observed the presence of IL-6, TNF-alpha, and IL-1beta that can be found in patients with SARS-CoV-2 in a conditioned medium from PSCV-treated MH-S or THP-1 cells (CMPSCV). These cytokines have been known to have pro-inflammatory and pulmonary fibrosis effects. IL-6 and TNF-α are proinflammatory factors but also inducers of pulmonary fibrosis. In the case of IL-6, it has been reported that IL-6 promotes the transformation of fibroblasts into myofibroblasts through the JAK/STAT3 signaling pathway^64^. TNF-α has been identified as a crucial cytokine in various inflammatory lung diseases. TNFα (TNFSF2) influences both structural cells, such as fibroblasts, and immune cells, like macrophages and T cells, promoting both pro-fibrotic and anti-fibrotic responses. TNF-α stimulated fibroblasts secrete lumican to promote fibrocyte differentiation^65^. A study by Maranatha et al. revealed a statistically significant correlation (p = 0.046) between elevated TNF-α serum levels in COVID-19 patients and the subsequent development of pulmonary fibrosis^66^.

In this study, the effects of apigetrin were evaluated using the SARS-CoV-2 complication mimic in vitro model. First, apigetrin was shown to block the activity between the binding spike S RBD and ACE2 receptor and to decrease spike protein expression (Fig. 3A,B). These results in apigetrin attenuated increase of inflammatory cytokine (IL-6, IL-1β, and TNF-α) levels on cytosol, and secretion by PSCV in MH-S and THP-1 cells (Fig. 5). Consistent with our data, apigetrin has been reported to lower TNF-α, IL-1β, and IL-6 production and mRNA expression in LPS-stimulated various cells^21,22,67^ and in mice with acute otitis media^24^. Similarly to our model, it has been reported that apigetrin inhibits IL-1α and TGF-β production induced by recombinant spike protein of SARS-CoV-2-stimulated RAW 264.7 cells^68^. Furthermore, apigetrin has been reported to possess potential inhibitory activity against SARS-CoV-2 3-chymotrypsin-like protease (main protease, Mpro) controlling this COVID-19 virus replication and is essential for its life cycle, according to computational analysis, including molecular docking and pharmacokinetic studies^69^. In this study, apigetrin is shown to attenuate MRC-5 cell proliferation, migration, expression of CTGF, COLA1, α-SMA, and HIF-1α induced by conditioned medium from PSCV-treated MH-S or THP-1 cells (CMPSCV) (Fig. 6).

## Conclusions

In summary, in this study, we evaluated the feasibility of an in vitro model of SARS-CoV-2 sequelae mimicking and used it to identify molecular mechanisms for HIF-1α targeting in SARS-CoV-2-associated diseases. Furthermore, apigetrin, a candidate drug for treating SARS-CoV-2 complications, was evaluated in this in vitro model. The PSCV particles treated MH-S and THP-1 cells showed increased expression of proinflammatory cytokines (IL-6, IL-1β, and TNF-α) and HIF-1α, consistent with other profiling data in SARS-CoV-2-infected patients have been reported. HIF-1α knockdown was used to determine the role of HIF-1α in this model, with HIF-1α knockdown decreasing increased proinflammatory cytokines levels and fibrosis-related protein levels in PSCV-stimulated MH-S and THP-1 cells and CMPSCV-cultured MRC-5 cells. Apigetrin inhibits the binding of the SARS-CoV-2 spike protein RBD to ACE2 SARS. Increased proinflammatory cytokines, HIF-1α, and spike protein levels were decreased by apigetrin in the SARS-CoV-2 ARDS mimic in vitro model. Apigetrin suppressed the increase of cell growth, migration, collagen synthesis, and fibrosis-regulatory proteins (CTGF, COLA1, α-SMA, and HIF-1α) in the PF model, CMPSCV-cultured MRC-5 cells. Thus, our data support that HIF-1α might be a novel target for SARS-CoV-2 sequela therapies, and apigetrin, a representative HIF-1α inhibitor, would have a potential value in regulating SARS-CoV-2 related diseases.

### Supplementary Information

Supplementary Figures.

## Data Availability

The data presented in this study are available on request from the corresponding author.
