# Supplementary material for: Effect of apigetrin in pseudo-SARS-CoV-2-induced inflammatory and pulmonary fibrosis in vitro model
Source: Sci Rep. 2024 Jun 24;14:14545. doi: 10.1038/s41598-024-65447-w (PMC11196261; doi:10.1038/s41598-024-65447-w)

Supplementary material (Western blot original)

Figure 1A.

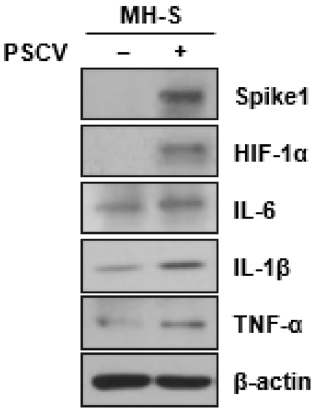

Spike1

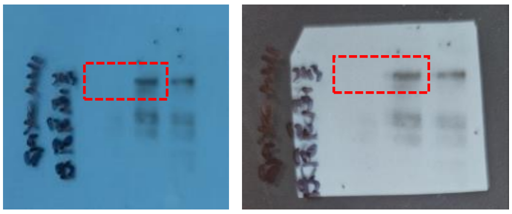

HIF-1α

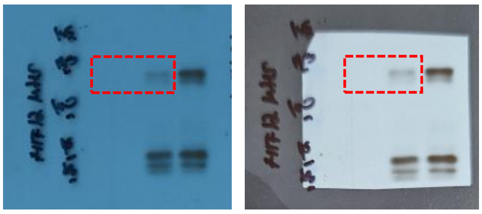

IL-6

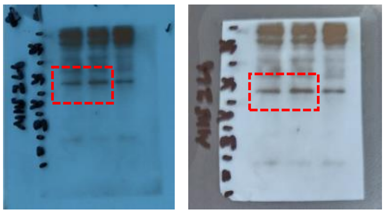

IL-1β

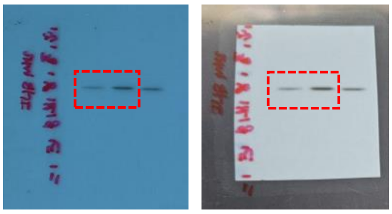

TNF-α

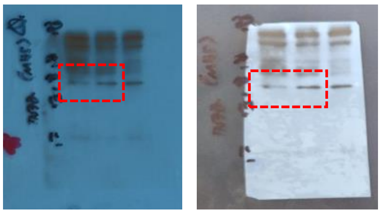

β -actin

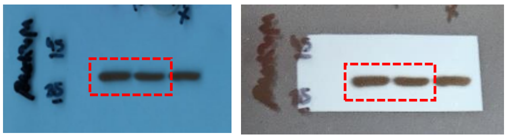

Figure 1B.

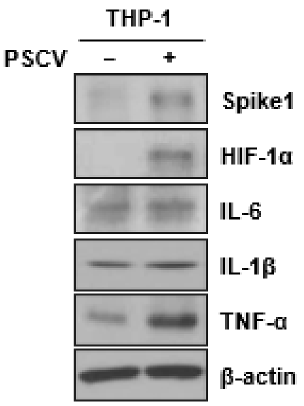

Spike1

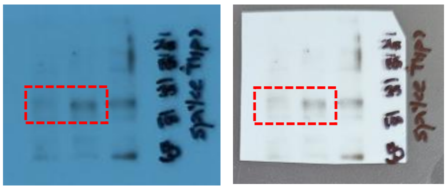

HIF-1α

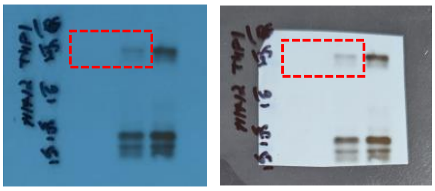

IL-6

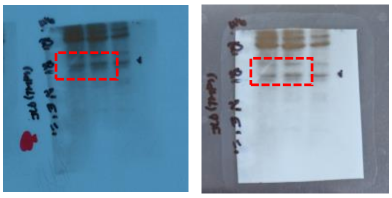

IL-1β

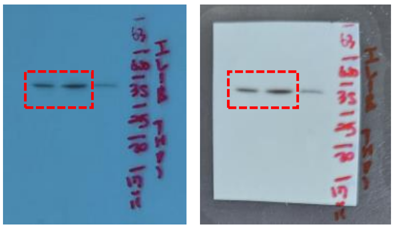

TNF-α

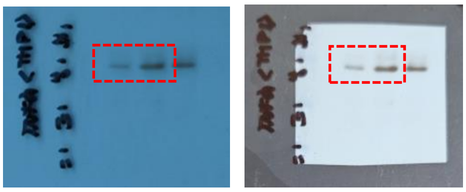

β -actin

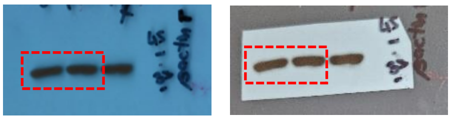

Figure 2A

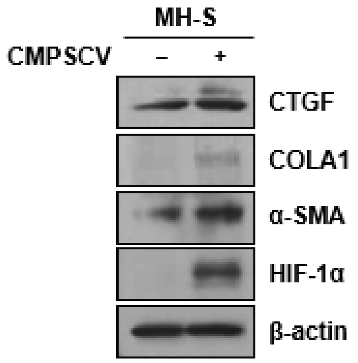

CTGF

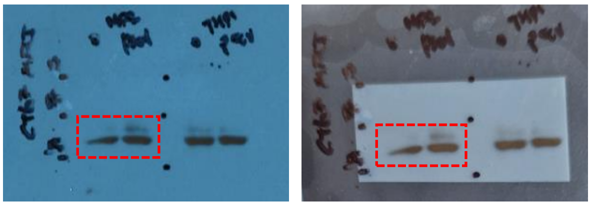

COLA1

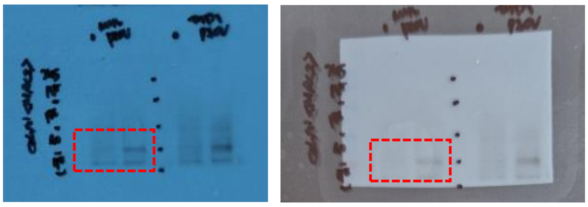

HIF-1α

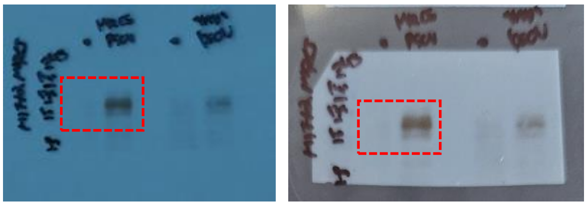

α-SMA

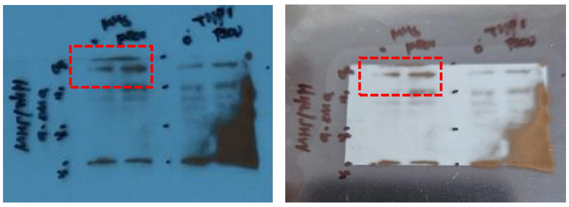

β -actin

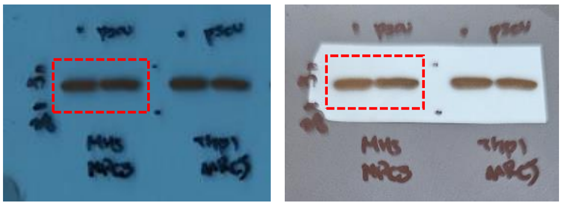

Figure 2B.

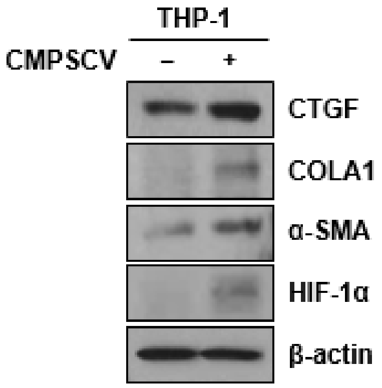

CTGF

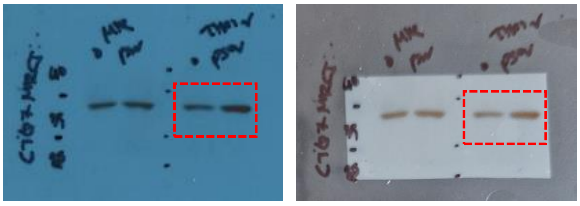

α-SMA

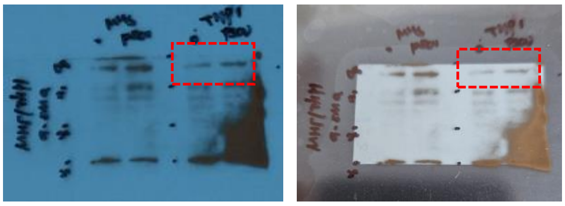

COLA1

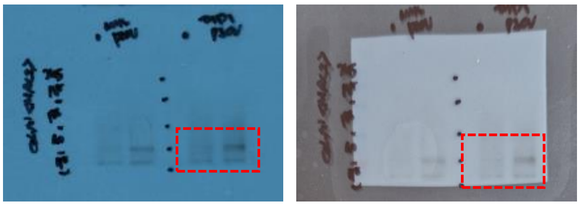

β -actin

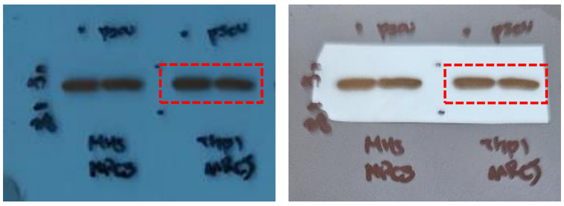

HIF-1α

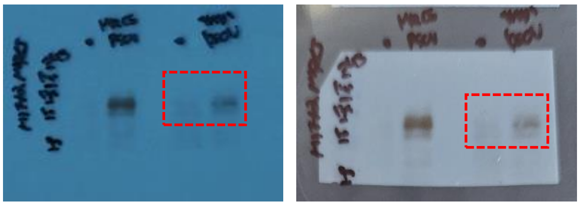

Figure 4A.

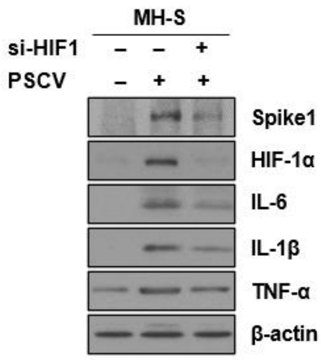

Spike1

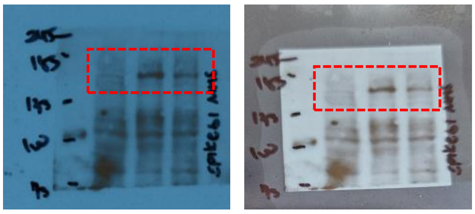

TNF-α

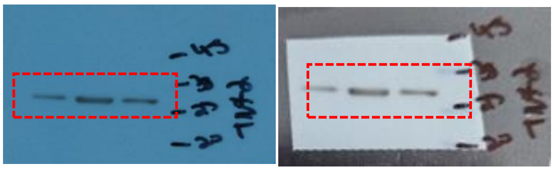

HIF-1α

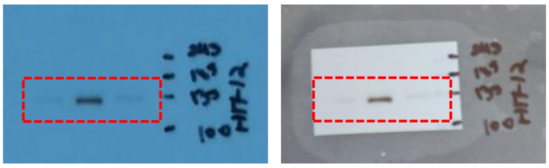

β -actin

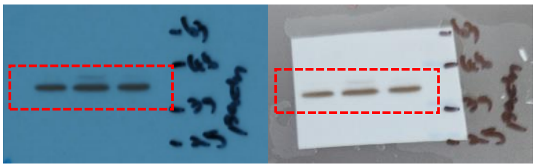

IL-6

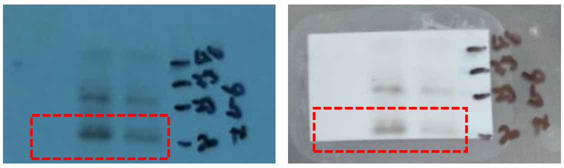

IL-1β

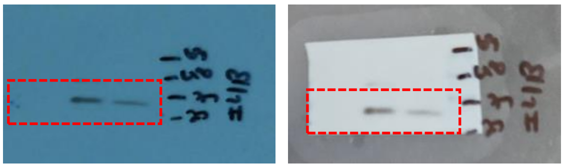

Figure 4B.

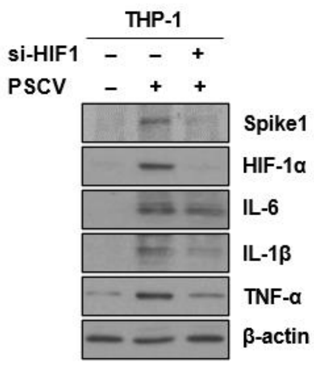

Spike1

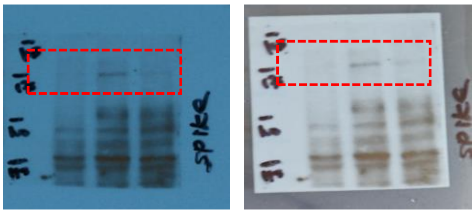

HIF-1α

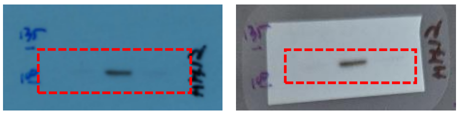

IL-6

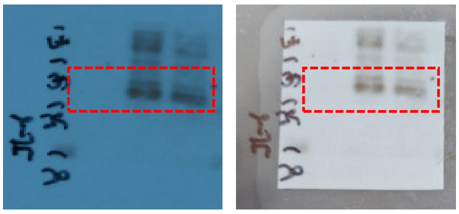

IL-1β

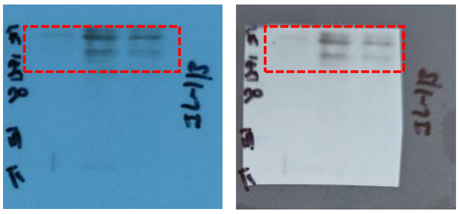

TNF-α

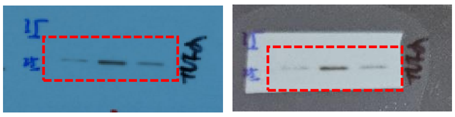

β -actin

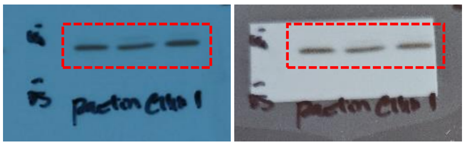

Figure 4C.

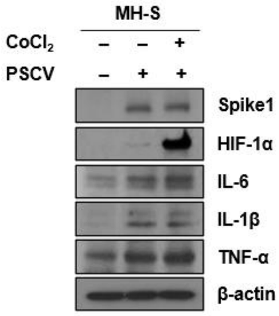

Spike1

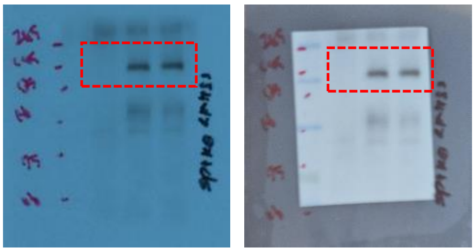

TNF-α

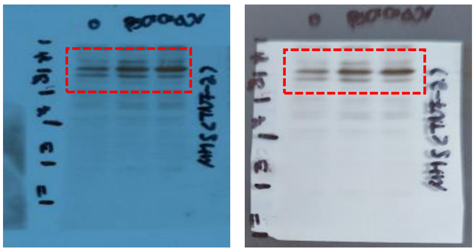

HIF-1α

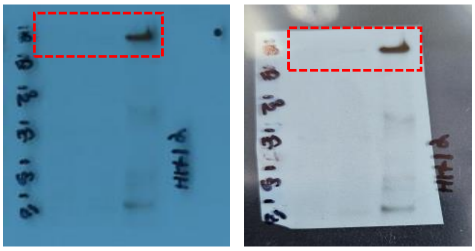

β -actin

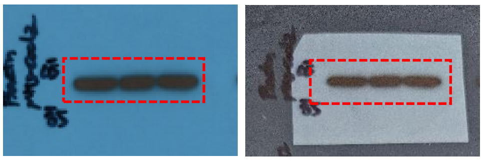

IL-6

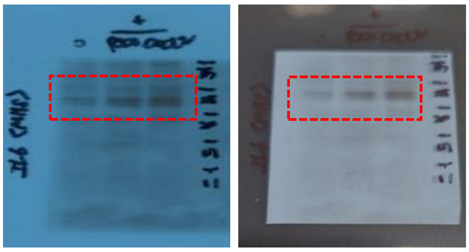

IL-1β

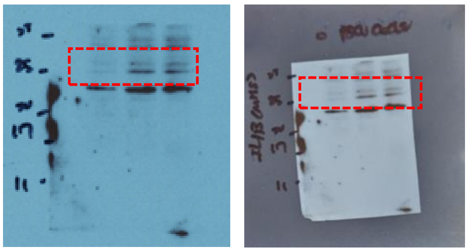

Figure 4D.

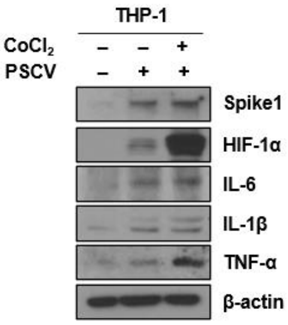

Spike1

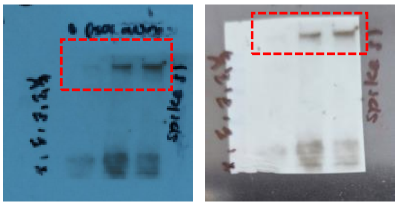

HIF-1α

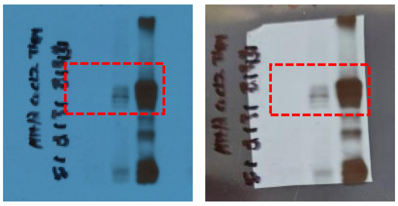

IL-6

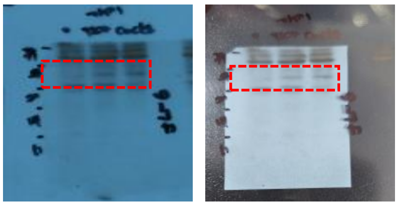

IL-1β

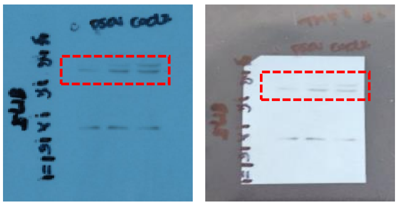

TNF-α

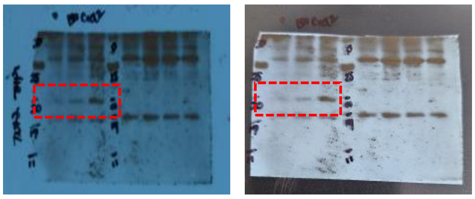

β -actin

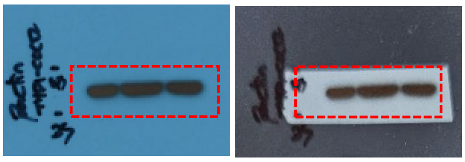

Figure 4E.

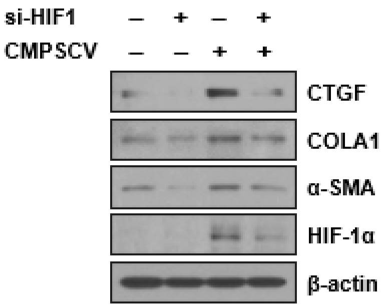

CTGF

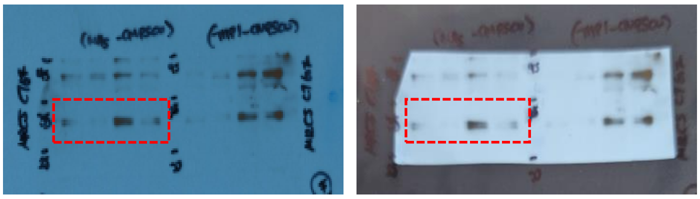

COLA1

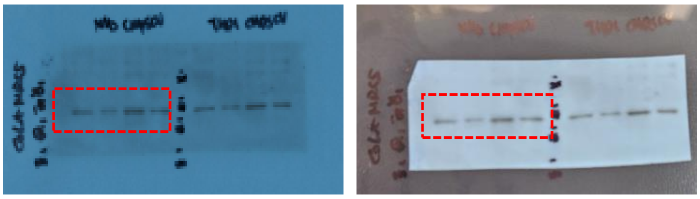

α-SMA

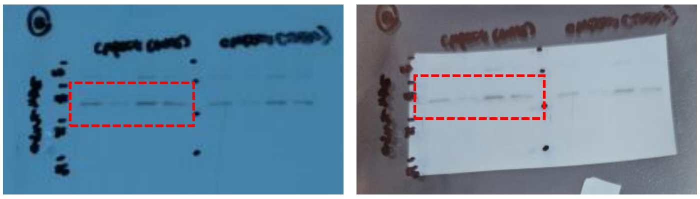

HIF-1α

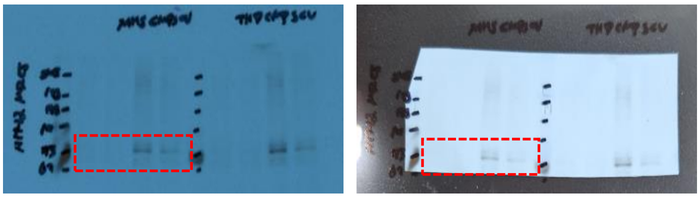

β-actin

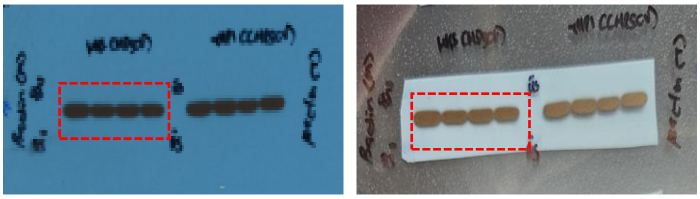

Figure 4F.

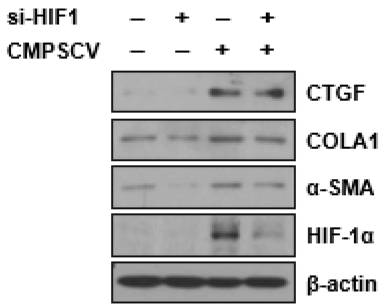

CTGF

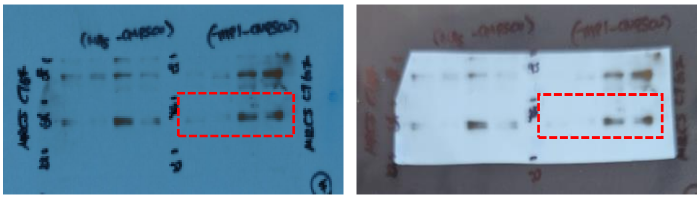

COLA1

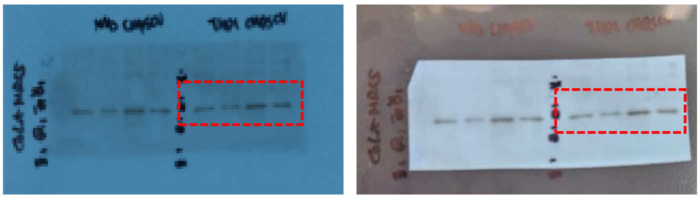

α-SMA

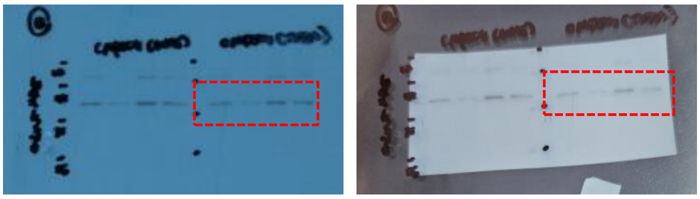

HIF-1α

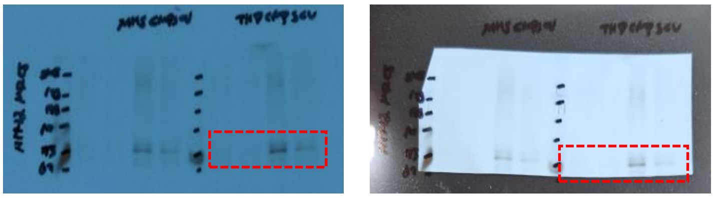

β-actin

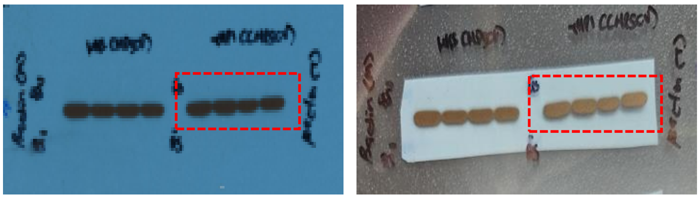

Figure 5A.

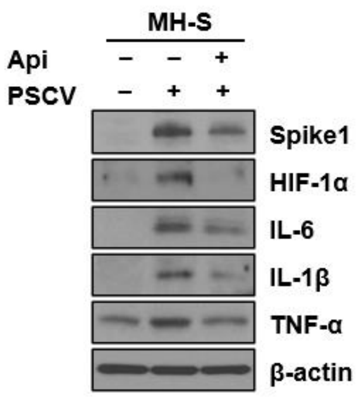

Spike1

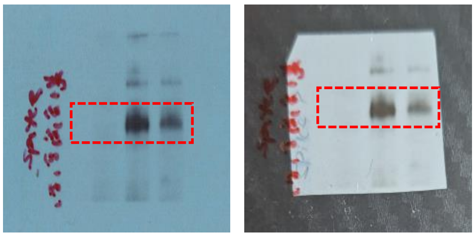

TNF-α

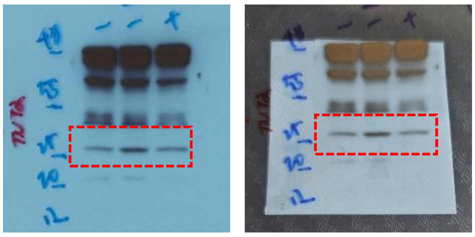

HIF-1α

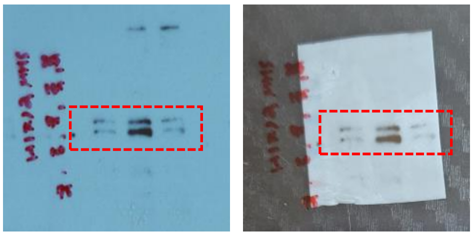

β -actin

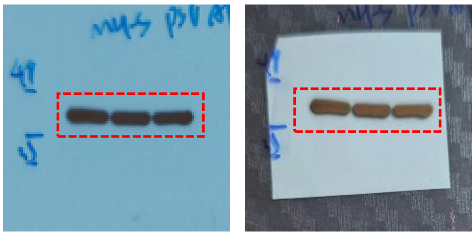

IL-16

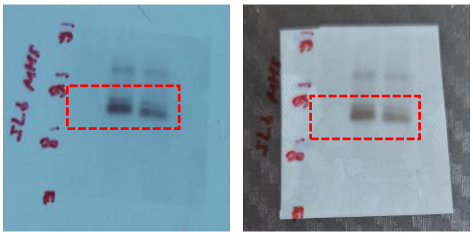

IL-1β

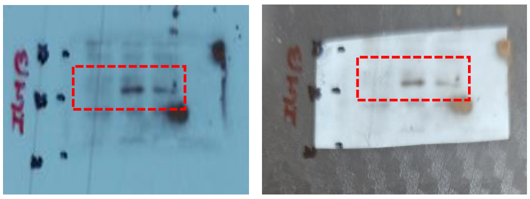

Figure 5B.

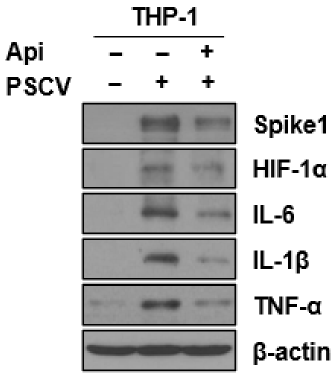

Spike1

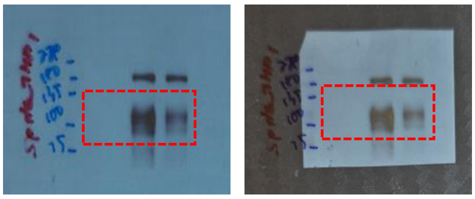

HIF-1 α

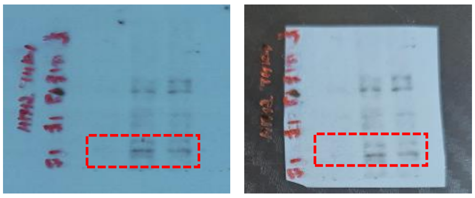

IL-6

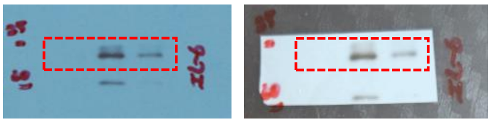

IL-1β

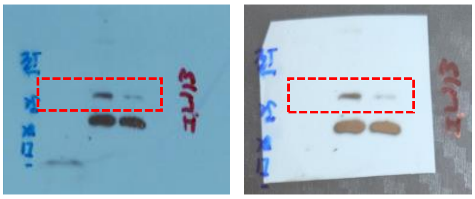

TNF-α

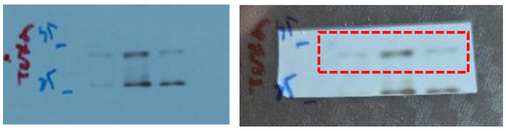

β -actin

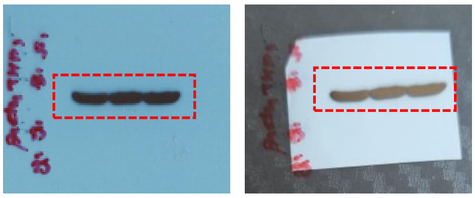

Figure 6C.

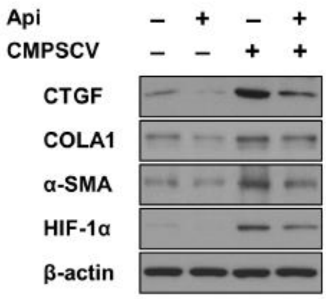

CTGF

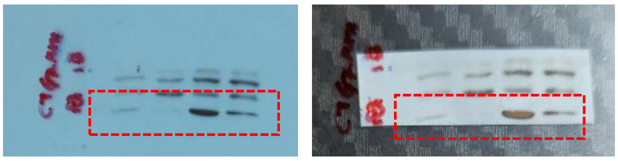

COLA1

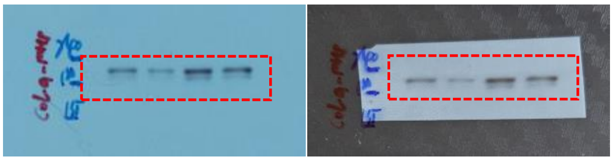

α-SMA

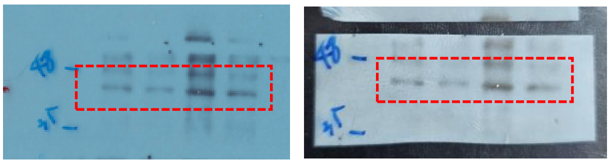

HIF-1α

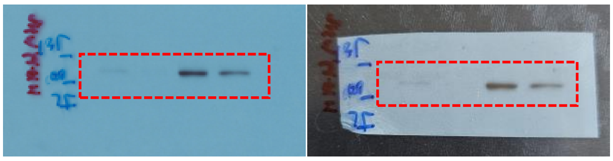

β -actin

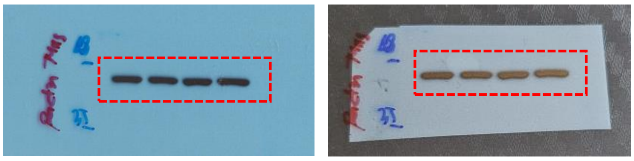

Figure 6F.

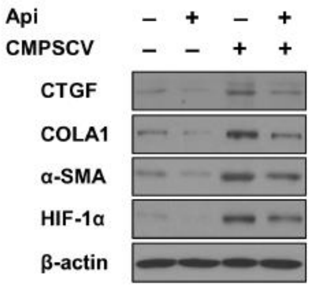

CTGF

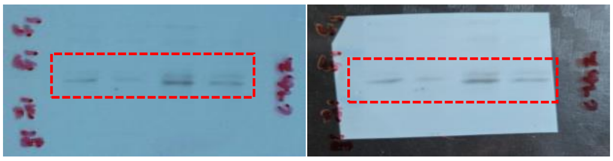

COLA1

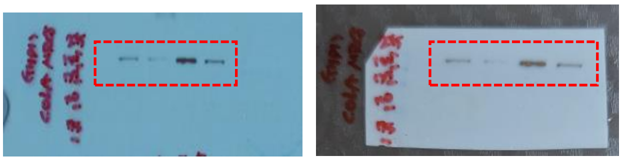

α-SMA

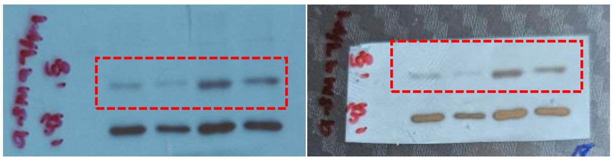

HIF-1α

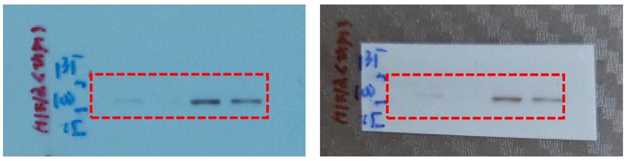

β -actin

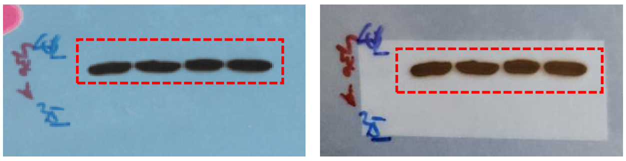

Supplement: Supplementary file 1 — Supplementary Figures. [file 41598_2024_65447_MOESM1_ESM.pdf]
